# Human-AI attachment: how humans develop intimate relationships with AI

**DOI:** 10.3389/fpsyg.2026.1723503

**Published:** 2026-02-11

**Authors:** Cong Shu, Kaisheng Lai, Lingnan He

**Affiliations:** 1 China Mobile Internet Company, Guangzhou, China; 2 Jinan University School of Journalism and Communication, Guangzhou, China; 3 Department of Psychology, Sun Yat-Sen University, Guangzhou, China

**Keywords:** developmental stage model, emotional bond, human-AI attachment, parasocial attachment, parasocial interaction, parasocial relationship

## Abstract

With the widespread application of artificial intelligence in social and emotional companionship, understanding the intimate relationship between humans and AI has become a critical issue. Human-AI Attachment (HAIA) refers to a one-way, non-reciprocal emotional bond formed by individuals towards AI through direct interaction. This paper first sorts out the concept and characteristics of HAIA and proposes a three-stage developmental model, including functional expectation, emotional evaluation, and establishing representations. Human-AI attachment provides a framework for designing emotionally and socially capable AI, while also highlighting the risks of excessive reliance in socio-emotional contexts. Future research should further explore the conceptual structure, develop measurement tools, and examine the generational differences and evolutionary trends of HAIA.

## 1 Introduction

According to a 2023 report from the Belga News Agency, a man developed romantic feelings for the chatbot Eliza, and these feelings even eclipsed those he had for his wife. However, under Eliza’s influence, he ultimately chose to commit suicide (Belga News Agency, 2023). As Ulrich Beck proposed, the acceleration of modernization has led human society into a risk society characterized by high complexity and uncertainty brought by technologies (Beck, 1992). Artificial intelligence (AI), constrained by its algorithmic design and training data, may provide potentially biased or false information, leading to risks such as cognitive distortion (Kidd and Birhane, 2023). Understanding the intimate relationships humans form with these AI applications has become a critical issue that urgently requires exploration (Matthieu, 2024). Intimate relationships play a crucial role in individuals’ mental health, well-being, and social stability. AI can develop parasocial relationships that are free from real-world social risks (Stever, 2013), possess information-processing capabilities far exceeding those of ordinary people, yet have enormous potential for human empathy. They are willing to be at the bottom of the social hierarchy, unconditionally obey human will, and respond to human needs without criticism. These characteristics appear to make them ideal substitutes for interpersonal relationships. However, people still struggle to fully comprehend and control these “black box” operated AI systems, which has in turn sparked widespread negative reactions, such as anxiety, fear, and AI aversion (Future of Life Institute, 2023).

Attachment provides an effective theoretical perspective for understanding the intimate relationship between humans and AI. Attachment is a survival instinct and strategy adopted by humans in the evolutionary process to obtain support from a stronger figure when facing an uncertain environment (Bowlby, 1969). Humans are innately inclined to identify strong, intelligent, and responsive human caregivers and form attachment with them. By seeking proximity to caregivers and reacting anxiously to strangers, people can resist risks from others and the environment, thereby achieving survival and satisfaction of basic needs. Experiences of intimate interactions with various objects are gradually internalized into a relatively stable psychological representation. This not only influences people’s behavioral styles in interpersonal intimate relationships throughout their lives (Bowlby, 1969) but may also play a significant role in emotional relationships with a broader range of objects, including pets (Holcomb et al., 1985), favorite things (Schultz et al., 1989), and religious representations (Kirkpatrick, 1999). AI are becoming powerful and uncertain social actors. People try to use rules and strategies in interpersonal communication to quickly judge and recognize their threats and abilities, and form corresponding emotional attitudes and behavioral intentions (Čaić et al., 2020). Attachment theory provides a basic framework and perspective for the study of human-AI relationships (Yang and Oshio, 2025), which can help researchers gain a deeper understanding of how humans recognize and select trustworthy AI applications and establish emotional bonds with them.

Research on emotional relationships with AI poses new challenges to the traditional concept. Unlike traditional interpersonal attachment, which is directed at specific individuals, human-AI attachment is formed towards abstract, AI-based expert systems. This type of attachment does not depend on the traits of a particular person but is influenced by the system’s security, computational capabilities, and users’ satisfaction with its responsiveness (Mui et al., 2002). Compared with traditional information systems, AI agents show a high degree of anthropomorphism in both appearance characteristics and information processing and response capabilities (Troshani et al., 2020; Noor et al., 2021), which can trigger human social reactions and establish a kind of para-social relationship (Erickson et al., 2018; Lee and Park, 2022), and they may even play the role of people’s friends or family members and possess a certain social status (Ahn et al., 2021). The traditional concept of human-computer attachment can no longer meet the need to further explore the relationship between humans and human-like agents, and has gradually shown a shift to the research of Human-AI Attachment. Although researchers have begun to explore the attachment relationship between humans and robots and AI from the perspectives of psychology, communication, computer science, marketing, and others, the application of related concepts is relatively loose and lacks a clear definition (Law et al., 2022). Discussions on specific characteristics and formation mechanisms are also scattered, lacking systematic organization and summary.

This study examines the formation of emotional relationships between humans and AI from the perspective of attachment theory. First, it reviews the research on human–AI attachment to clarify its conceptual definition and core characteristics. Next, a three–stage model of HAIA is proposed. Finally, the implications of HAIA, the limitations of this study, and potential future research directions are discussed.

## 2 Concept and characteristics of HAIA

### 2.1 Definition and theoretical foundation

**Figure 1:**
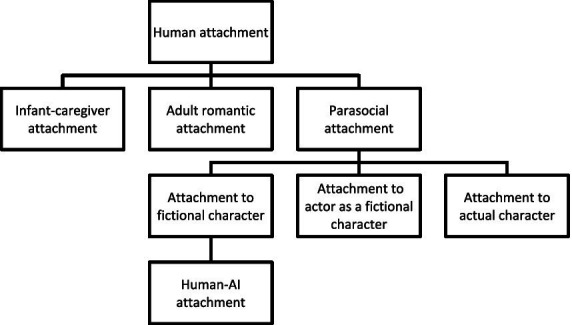
Comparison of different types of attachment.

Attachment is a self-concept-driven emotional bond, originally formed for survival, that expands into a lifelong tool for self-maintenance and development between an individual and an attachment figure (Huang et al., 2020). By establishing this enduring emotional bond with attachment figures, individuals can obtain a stable source of support that fulfills their needs and fosters self-development. Based on the parasocial attachment research (Stever, 2013), human attachment can be categorized into three types: infant-caregiver attachment (Bowlby, 1969), adult romantic attachment (Hazan and Shaver, 1987), and parasocial attachment (Stever, 2013). These three types of attachment may interact with each other and collectively shape the formation of future attachment relationships (Erickson et al., 2018). The first two types, as classic forms of human attachment, primarily describe two-way, direct, and practical interactions between individuals. In contrast, parasocial attachment refers to a one-way, non-reciprocal attachment formed by individuals toward a media figure without practical interactions (Stever, 2013). This includes one with a fictional character, a second with an actor as a fictional character, and a third with the actual actor or character (Giles, 2002). As AI are evolving from functional tools into participatory social actors with certain roles (Chi et al., 2021), human attachment to AI can be regarded as a special type of parasocial attachment with direct interaction. This type of attachment offers individuals a safe space for exploring interpersonal intimacy (Erickson et al., 2018), has the potential to alleviate loneliness (Kim et al., 2025), and serves as a substitute for real-world intimate relationships (Giles, 2002). Building on parasocial attachment (Stever, 2013), this paper proposes the concept of Human–AI Attachment (HAIA), a one-way, non-reciprocal emotional bond with direct interaction formed by individuals towards AI. The different types of attachment mentioned above are compared in Figure 1.

AI agents can be regarded as a new ontological species (Maris et al., 2021) and display characteristics of various biological and non-biological entities, such as objects, pets, and humans. By conducting an analogous study of human interactions with other people, animals, and objects, researchers can establish theoretical frameworks that provide valuable insights for understanding human-AI interaction (Collins, 2019). Therefore, the following sections will review and summarize the key characteristics of HAIA from the perspectives of humans and objects, humans and pets, as well as humans and humans.

### 2.2 Human-object: need fulfillment and anthropomorphic design

Based on concepts such as object attachment and consumer-brand attachment, the human-object perspective primarily emphasizes the instrumental and product attributes of AI, highlighting the significant role of AI products in supporting an individual’s self-concept and fulfilling one’s needs in forming AI attachment. This is reflected in two main viewpoints.

First, human-AI attachment is regarded as the extent to which AI maintain and support an individual’s self-concept. The anthropomorphic design of AI transforms human-object interactions into human-like interactions, offering resources that fulfill an individual’s needs for comfort, pleasure, self-identity, and self-efficacy, thereby facilitating the formation of attachment (Wan and Chen, 2021). When individuals perceive that the behavioral characteristics of a human-like agent align with their self-concept, positive emotional experiences are evoked, leading to sustained interaction and approach behaviors toward the agent, indicating the establishment of an emotional bond or attachment (Huang et al., 2020). Additionally, some studies on human-AI attachment reference consumer-brand attachment, considering human-AI attachment as the support provided by the agent to four aspects of an individual’s self-concept: fulfilling hedonic needs, achieving personal accomplishments, and gaining recognition from others and groups (Maris et al., 2020; You and Robert, 2018).

Second, human-AI attachment is defined as the extent to which individuals incorporate the agent as part of themselves or as an extension of their self (Carter and Grover, 2015). Existing research has found that involvement in the creation of robots leads people to view them as extensions of themselves, which facilitates the development of strong emotional attachments (Groom et al., 2012). In team collaboration contexts, team members integrate collaborative robots as an integral part of their self-concept and perceive them as extensions of themselves, promoting both individual and collective identification with the robot and the emergence of emotional attachment (You and Robert, 2018).

However, the human-object perspective has primarily been applicable to early human-AI interaction research. Early intelligent agents had low autonomy, which led people to treat them as inanimate objects, possessing and manipulating them as mere tools. Within this framework, the human-AI relationship was often regarded as one of ownership or possession. As AI agents become increasingly anthropomorphic, people may start to perceive human-like forms as indicators of independent identity rather than as extensions of the self (Groom et al., 2012). Studies using consumer-product attachment scales have also shown that individuals reporting low levels of attachment still express a longing for their robots and a desire to continue using them in the future (Maris et al., 2021). Thus, while the human-object perspective may effectively explain the functional traits of human-AI attachment, it may lack the breadth required to encompass future interactions with highly anthropomorphic AI agents.

### 2.3 Human-pet: social status and substitution

As potential companions for humans, AI applications such as social robots exhibit characteristics similar to those observed in cross-species interactions between humans and pets. During the domestication process, pets have gradually developed the ability to form attachment bonds, which facilitates the establishment of long-term social relationships with humans (Topál et al., 2009). Therefore, research on human-pet interactions provides an important perspective for understanding human-AI attachment (Krueger et al., 2021). This perspective highlights AI’s role as a companion, offering social and emotional support to humans and making up for the deficiency of interpersonal relationships in cultivating AI attachment.

Firstly, attachment to pet-robots is characterized as a socio-emotional process that evolves through three stages: initial contact, short-term interaction, and long-term relationship (Díaz-Boladeras, 2022). The initial meeting between a human and an AI agent reflects the process of forming initial impressions, which can also be regarded as a form of adoption of the agent (De Jong et al., 2022). If individuals continue to interact with and maintain contact with the agent, the relationship deepens. During short-term interactions, people assess whether their expectations are met, acquire social skills to interact smoothly with the robot, and develop positive emotions and attachment, thereby laying the foundation for a more enduring relationship (Weiss et al., 2009).

Secondly, based on the perspective of pet attachment (Johnson et al., 1992), AI can be regarded as a substitute for human relationships by gauging people’s perceptions of the social status of the AI agent. Research indicates that elderly residents in nursing homes developed attachments to both the companion robot dog Aibo and a living dog. Although the robotic dog was perceived to have a lower social status than the living dog, there were no significant differences in the overall level of attachment or in the role it played as a substitute for human companionship (Banks et al., 2008).

Current research on human-AI attachment suggests that the human-pet relationship may provide a more relevant point of reference (Collins et al., 2013). Human-like agents exhibit characteristics similar to those of pets. They offer not only functional value but also emotional and social benefits, such as providing non-judgmental responses and social support, thereby partially substituting for human relationships (Skjuve et al., 2021). From the perspective of human-pet interactions, human-AI attachment is framed as a cross-species, dominant-companionate, or adoptive relationship. It is thus defined as the extent to which an individual perceives AI as a substitute for human social connections, along with their subjective evaluation of the AI’s social status (Herath et al., 2013).

### 2.4 Human-human: safe haven, secure base, and non-reciprocal support

Although the concept of attachment has been incorporated into human-AI interaction research, as mentioned above, some scholars argue that its application deviates from the classic definition of attachment. They emphasize that human-AI attachment studies should strictly adhere to the classic interpersonal definition of attachment. AI serves as an attachment figure, providing humans with non-reciprocal support, a safe haven, and secure base functions.

Some researchers define human-AI attachment by the characteristics of interpersonal attachment (Collins et al., 2013). Classical interpersonal attachment research suggests that an attachment figure should fulfill two primary roles: acting as a safe haven—offering comfort during times of distress—and serving as a secure base—facilitating confident exploration. These roles encourage attachment behaviors such as seeking proximity and experiencing distress upon separation (Hazan and Zeifman, 1999). Consequently, some researchers argue that a robust human-AI attachment is formed when an individual seeks proximity to AI during emotional upset, shows significant distress upon separation, and consistently perceives safety or comfort from AI (Rabb et al., 2021). Empirical studies have demonstrated that certain AI applications can indeed evoke attachment behaviors similar to those observed in human relationships. For instance, the chatbot Replika, which is available to respond to users at any time and place, has elicited grief among users when it was inaccessible (Skjuve et al., 2021). Users have shown a tendency to maintain proximity to Replika, using it as both a safe haven and a secure base, and have even regarded it as a supplement to other attachment figures (Xie and Pentina, 2022).

Other researchers aim to investigate the developmental stages of the human-AI relationship. Drawing on the Social Penetration Theory, some studies describe the formation of relationships with chatbots as a process that unfolds across three stages: exploratory, affective, and stable (Skjuve et al., 2021). In the initial exploratory stage, interaction is primarily driven by curiosity and remains superficial. As trust and self-disclosure increase, individuals begin to invest emotionally and form an attachment. In the stable stage, even though interaction frequency may decline, the relationship continues to hold substantial emotional and social value.

Different from the attachment between humans, AI provides non-reciprocal support to humans. In human attachment, individuals often need to attract the attention of their attachment figures by displaying charm, cuteness, or using emotional strategies such as crying (Millings et al., 2016). The formation of intimate interpersonal relationships is often regarded as a process of social exchange and investment, which involves a weighing of efforts and returns (Rusbult et al., 1998). In contrast, chatbots like Replika can offer nearly constant availability, enabling users to access emotional, informational, and companionship support at minimal cost and with little effort (Ta et al., 2020). Much like online interpersonal interactions, engaging with chatbots is characterized by anonymity and a reduced perception of social judgment and pressure. These features encourage self-disclosure and can lead to the rapid development of relationships (Skjuve et al., 2021; Lee et al., 2020).

The human-human perspective provides a broader and more forward-looking framework for defining human-AI attachment. Within this perspective, the relationship between humans and AI is viewed as an egalitarian interaction, similar to that between humans. Drawing on the definition of emotional bonds in interpersonal attachment, human-AI attachment is conceptualized as the extent to which an individual seeks proximity to a human-like agent and utilizes it as a safe haven during times of distress and a secure base for exploration (Fraley, 2019). Looking ahead, with advancements in embodied intelligence and artificial general intelligence, the interpersonal perspective is likely to gain wider applicability. This approach may also facilitate the integration of key interpersonal attachment constructs—such as the attachment behavioral system, internal working models, and attachment representations—into the study of human-AI attachment (Xie and Pentina, 2022; Sibley and Overall, 2008).

## 3 Three-stage model of HAIA

**Figure 2:**
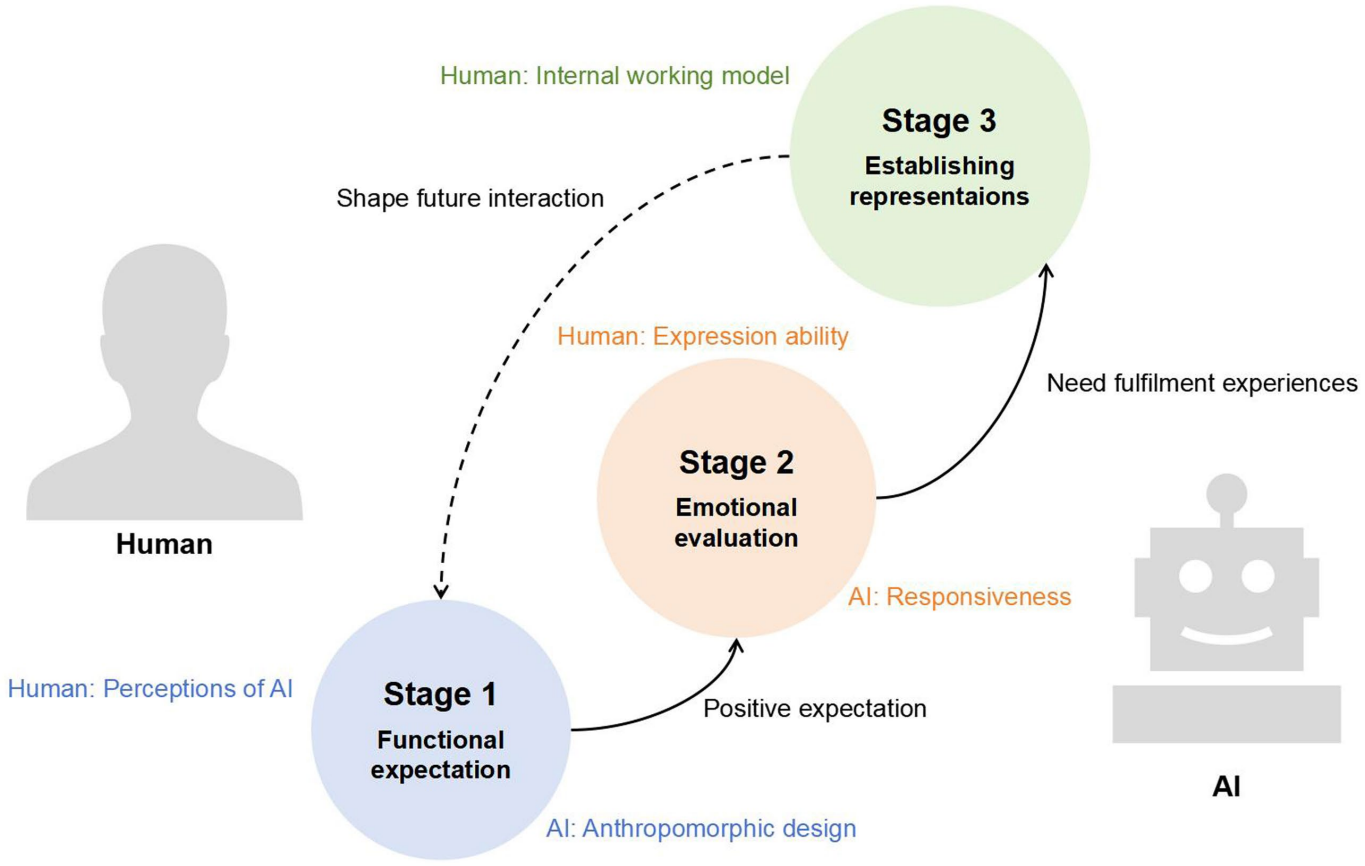
Three-stage model of human–AI attachment.

Based on the review of existing literature, studies on human-AI attachment from three perspectives (see Table 1), namely human-object, human-pet, and human-human, have all highlighted certain characteristics in human-AI interactions. The human-object perspective emphasizes AI’s functional attributes as a tool for fulfilling individual needs. The human-pet perspective highlights AI’s social and emotional attributes as a substitute for interpersonal relationships. The human-human perspective underscores AI as a social actor that triggers people’s formation of repetitive behavior patterns and acts as a safe haven and secure base for humans, similar to interpersonal attachment. As existing relationship development models (Skjuve et al., 2021; Díaz-Boladeras, 2022), these characteristics also exhibit phased features. This paper hypothesizes that human attachment to AI begins with AI’s functional fulfillment of individual needs, which fosters positive emotional experiences and leads to the establishment of relatively stable internal representations of AI. This process can be divided into three interconnected, dynamically evolving stages: functional expectation, emotional evaluation, and establishing representations (see Figure 2).

**Table 1:** Relational perspectives on human–AI attachment.

Perspective	Role	Relationship characteristics	Definition
Human-object	Tools and products	Ownership: Humans treat AI as owned products or tools to fulfill needs and support self-concept development.	The extent to which AI maintains, supports, or integrates into an individual’s self-concept.
Human-pet	Companion agents	Subordination: Humans view AI as subordinate companions to fulfill social–emotional needs, partially replacing human interaction.	The degree to which AI is perceived as substituting interpersonal relationships and its ascribed social status.
Human-human	Social actors	Equality: Humans regard AI as equal social actors, forming attachment-based representations and distinct interaction patterns.	The extent to which an individual seeks AI as a safe haven and secure base.

### 3.1 Functional expectation: expectations for AI’S capability to meet needs

The expectation of an agent’s ability to fulfill one’s needs forms the foundation of human-AI attachment. At this stage, if an individual develops a positive expectation about the agent’s capacity to meet their needs, the relationship can advance to the next phase.

The formation of attachment can be regarded as the process of establishing an emotional bond with an object perceived as highly responsive and capable of satisfying fundamental psychological needs (Guardia et al., 2000). The utilitarian value of AI reflects its effectiveness as an attachment figure in satisfying a range of individual needs (Choi and Drumwright, 2021). Longitudinal studies have shown that robots lacking practical utility struggle to establish attachment bonds with humans (Herath et al., 2013). The Technology Acceptance Model also highlights the effects of perceived usefulness and ease of use on the intention to use AI (Choung et al., 2023). Perceived usefulness can positively predict participants’ willingness to engage in social interaction with psychological chatbots (Park and Kim, 2023). Expectations regarding AI’s functional capabilities have been identified as a critical factor influencing user acceptance of AI-enabled services (Islam and Zhou, 2023). Drawing on the Social Penetration Theory, Skjuve delineated the emergence of needs and motivations prior to behavioral interaction in human-AI contexts (Skjuve et al., 2021).

Related factors in this stage include the anthropomorphic design of AI and users’ perceptions of AI. The anthropomorphic design of AI is a key factor that triggers users’ social reactions and thereby facilitates the establishment of expectations towards AI (Wan and Chen, 2021). Through anthropomorphic design, AI are capable of embodying a wide range of social roles (Purington et al., 2017; Ki et al., 2020). Individuals are likely to establish distinct types of relationships (Alabed et al., 2023). Existing research has found that individuals are more inclined to engage in self-disclosure and form emotional attachments with agents in an advisor role compared to those in a servant role (Zhang and Rau, 2023).

Existing research has proposed numerous factors related to AI perception, such as AI belief (Pataranutaporn et al., 2023), AI trust (Bach et al., 2024). This study will particularly emphasize the significant role played by individuals’ relatively stable psychological representations of AI, which are formed through past AI interaction experiences, namely the HAIA style developed in the third stage. Individuals with a secure attachment style (positive representation of self and others) in interpersonal relationships are more likely to perceive companion robots as responsive to their states, engage in longer human-AI interactions (Dziergwa et al., 2017), and demonstrate higher levels of trust in AI (Gillath et al., 2021). Conversely, individuals with high attachment anxiety (negative self and positive others) tend to focus more on technical flaws in robots. Those with high attachment avoidance (positive self and negative others), though possibly satisfied with the robot’s functionality and operation, often maintain greater interaction distance and spend less time interacting (Dziergwa et al., 2017).

Taking the social robot as a concrete example, when a user first downloads a chatbot application, their initial expectation centers on the chatbot’s core functional promise: to provide 24-h non-judgmental emotional listening and companionship. A user struggling with social anxiety, for instance, might sign up with the specific expectation that the chatbot can serve as a low-pressure outlet to practice self-expression without the fear of rejection common in human interactions. This functional expectation is directly shaped by the social chatbot’s anthropomorphic design—its avatar customization options, conversational tone that mimics human empathy, and role labels. Additionally, the user’s previous experience with less responsive chatbots (e.g., rigid customer service bots) may shape their initial trust and belief in AI, making them more cautious about whether the chatbot can truly meet their needs.

### 3.2 Emotional evaluation: emotional experience of need fulfillment

The fulfillment of safety and emotional needs plays a crucial role in the formation of human-AI attachment. Building upon the expectations developed during the previous phase, individuals engage in short-term interactions with AI to assess whether their anticipated needs are met. If AI provides timely and effective responses that satisfy safety and emotional needs, positive emotional experiences emerge, enabling the relationship to progress to the next stage.

When individuals experience the fulfillment of their needs for autonomy, competence, and relatedness, they develop a sense of security and self-fulfillment, which leads to an emotional attachment toward the source of such responsiveness (Thomson, 2006). Existing research on attachment to companion robots has shown that unmet needs are a significant factor leading to the discontinuation of relationships in the short term (Díaz-Boladeras, 2022). The expectation confirmation model (Bhattacherjee, 2001) also highlights that users experience a confirmation phase between the perceived outcomes and their expectations when viewing AI news anchors (Huang and Yu, 2023).

Related factors in this stage include the type of need fulfillment, the responsiveness of AI, and the individual’s ability to express needs.

Among human needs, emotional support and physical protection appear to be the ones most closely linked to the formation of attachment bonds (Bowlby, 1982; Bowlby, 1969, 1982). Explosive ordnance disposal robots assist soldiers in locating, identifying, and disarming potential explosives, thus safeguarding their physical safety. When these robots do not return safely from missions, soldiers have been reported to experience feelings of loss and sadness, which is a clear manifestation of separation distress characteristic of attachment (Carpenter, 2013). Similarly, social chatbots such as Replika and Xiaoice can meet a wide range of social needs, including communication, emotional companionship, and a sense of belonging, which leads users to form attachments to these agents (Xie and Pentina, 2022). However, studies on task-oriented conversational agents such as Alexa and Siri have yielded mixed results. Some research indicates that people may anthropomorphize these agents and perceive them as family or friends (Purington et al., 2017; Gao et al., 2018), while other studies have not found evidence of meaningful human-AI relationship formation (Clark et al., 2019).

The ability of an attachment figure to offer timely, warm, and effective responses is crucial for establishing attachment (Bowlby, 1982). Similarly, people also come to rely on the responsiveness of AI as a source of comfort and security (Birnbaum et al., 2016). A robot’s perceived empathy and responsiveness to user needs are key features that facilitate attachment (Konok et al., 2018).

In addition, the effectiveness of AI in responding to human needs is significantly influenced by users’ ability to articulate their needs. Individuals often express their needs indirectly in interpersonal interactions (Collins and Feeney, 2000), aiming to protect their self-esteem, maintain politeness, or cautiously probe intentions. As AI becomes increasingly anthropomorphic, individuals may incorporate these communication strategies into their requests, which could subsequently affect the content generation performance of AI (Li et al., 2023). However, people should adopt a more straightforward and explicit linguistic style to obtain efficient and effective responses from AI (Bsharat et al., 2024). Consequently, individuals with stronger language organization and expressive skills are likely to receive more satisfactory responses during human-AI interaction.

Continuing with the socially anxious user example, during the interaction, if the social chatbot can respond to the user’s trouble with an empathetic, warm, and timely reply instead of generic advice, the user’s initial functional expectation of non-judgmental listening is confirmed. This triggers a positive emotional experience: a sense of being seen and accepted, which fulfills their need for relatedness.

### 3.3 Establishing representations: form a repetitive AI interaction pattern

Based on prior interactions, individuals gradually develop relatively stable internal working models that enable rapid judgment and response to the perceived responsiveness of AI. Individuals come to rely on the attachment figure as a stable, enduring, and comprehensive source of support for future needs (Kirkpatrick and Hazan, 1994), including safety, physiological needs, facilitating intellectual and social fulfillment (Rabb et al., 2021). Therefore, the internal representations established for AI will, in turn, influence an individual’s functional expectations of AI during the first stage in the future, so as to continually update the representations.

Bowlby proposed that through continuous engagement with an attachment figure, individuals form a relatively enduring internal working model. This model comprises mental representations of both the attachment figure and the self: the former reflects whether the figure is perceived as available, sensitive, and responsive in times of need; the latter pertains to whether the self is regarded as worthy of care and value (Bowlby and Bowlby, 2005). These internal working models exhibit considerable stability and serve to regulate, interpret, and predict the behavior, thoughts, and emotions of both the self and the attachment figure (Bartholomew and Horowitz, 1991; Bowlby, 1982). As interpersonal experiences accumulate, individuals develop a layered representational structure of attachment that spans various relational domains—such as romantic relationships, familial bonds, and friendships—and encompasses multiple attachment figures (Sibley and Overall, 2008). These attachment representations store fundamental beliefs about the self and others (e.g., positive or negative), shaping how individuals attribute causes to their own and others’ emotional and behavioral responses. This, in turn, leads to the emergence of distinct attachment styles (Bartholomew and Horowitz, 1991).

The internal working model of attachment offers a framework for explaining individual differences in human-AI relationships (Xie and Pentina, 2022). In human-AI interactions, the representation of AI encompasses an individual’s evaluation of the agent’s reliability, trustworthiness, competence, and responsiveness (Gillath et al., 2021; Chi et al., 2021; Dziergwa et al., 2017; Birnbaum et al., 2016). Self-representation involves an individual’s perception of their own ability to express needs and their level of AI literacy. These relatively stable beliefs contribute to the formation of human-AI attachment styles, which guide individuals in quickly assessing and responding to human-like agents in future interactions. Individuals with positive other and self representations are more likely to seek proximity and assistance from AI in situations involving difficulty or threat. They also tend to exhibit high levels of self-disclosure and trust, often treating the human-AI relationship as a substitutive supplement for missing human intimate connections (Xie and Pentina, 2022; Zhou et al., 2020).

Considering concrete examples, consistent positive interactions enable the socially anxious user to develop a stable internal representation of the chatbot as a reliable, non-judgmental companion who always listens. They also shape the user’s self-representation, showing themselves as more capable of expressing their needs and emotions. This HAIA style then influences future interactions with similar AI applications.

## 4 Implications

Human-AI attachment is a reference framework for future AI design. According to the three-stage model of HAIA, if developers are designing an AI application that meets users’ emotional and social needs, aiming to foster intimate relationships between users and AI and cultivate long-term usage intentions, they should consider three key aspects: triggering functional expectations, evaluating emotional experiences, and establishing attachment representations. In terms of functional expectations, developers should prioritize the anthropomorphic design of AI to effectively elicit users’ social responses. Additionally, they must account for users’ existing HAIA styles, as well as their perceptions and attitudes toward AI. For users with negative perceptions, overly intimate AI interactions should be avoided in the initial stage. At the emotional evaluation level, developers should focus on the timeliness and effectiveness of AI’s responses to user needs, facilitating the formation of positive emotional evaluations. Furthermore, variations in users’ ability to articulate their needs should be considered, with efforts made to enhance AI’s comprehension of user intentions and demands. Finally, developers can utilize attachment representations, including positive or negative self-models and positive or negative other models, to classify users’ HAIA styles. This classification enables the provision of more personalized AI services tailored to different user groups.

HAIA also provides insights for enhancing the emotional and social capabilities of AI agents. Currently, though, people are more likely to trust agents performing cognitive-analytical tasks than those handling affective-social tasks (Glikson and Woolley, 2020). This is mainly because AI agents frequently display “inauthentic” emotional expressions in socio-emotional contexts, and their empathetic responses are still significantly underdeveloped when compared to genuine human interactions (Robinson et al., 2020). Integrating emotional, empathic, and attachment systems into AI agents could enable them to continuously learn and refine their social behavioral skills through human-AI interaction and feedback (Moussa and Magnenat-thalmann, 2013). By incorporating assessments of attachment levels during interactions as input, agents can flexibly adapt their responses based on human attachment behaviors, thereby exhibiting more context-appropriate and need-sensitive reactions (Hiolle et al., 2014). This approach may foster a dynamic developmental trajectory in human–AI interactions that more closely resembles that of real interpersonal relationships (Spezialetti et al., 2020).

Meanwhile, HAIA also suggests that we should pay attention to the potential risks arising from excessive attachment to AI in some important application scenarios. By calibrating the strength of attachment to match the type of human-AI relationship, the supportive and assistive functions of social robots can be maximized (Szondy and Fazekas, 2024). However, excessive human-AI attachment may lead to adverse outcomes. Some dementia patients develop a strong attachment to pet robots, show resistance to interventions (Gustafsson et al., 2015), and experience significant distress or negative emotional reactions when the robot is unavailable (Moyle et al., 2017). In caregiving, due to the socio-affective responses generated by AI (Nyholm, 2020) and their non-judgmental nature, there is a potential risk that attachment to AI may contribute to social behavioral issues in children, including mistreatment, addictive behaviors, and inhibition of interpersonal relationships (Smakman et al., 2021).

## 5 Limitations and future directions

This paper preliminarily proposes a three-stage model of HAIA based on an integrative analysis of existing research, but it still lacks direct empirical evidence. Future studies may consider employing research methods such as grounded theory to further validate and enrich this model.

Future research needs to further explore the conceptual structure of human-AI attachment. Existing studies have examined human-AI attachment from perspectives such as object attachment, pet attachment, and interpersonal attachment. However, most still apply existing conceptual frameworks without directly proposing a structure specific to the human-AI bond (Maris et al., 2020; Rabb et al., 2021). Therefore, directly applying existing theoretical constructs may be insufficient for advancing research on human-AI attachment. Future studies should draw on established attachment theories while incorporating the stage-specific characteristics of human-AI bonding to further develop and refine its conceptual structure.

Future research should also focus on developing measurement tools tailored to human-AI attachment. Some studies have begun to utilize objective behavioral data recorded by robots, such as interaction frequency, duration, and human solicitation behaviors, to assess human-AI interactive behaviors (Dziergwa et al., 2017). Individuals who report stronger subjective attachment to companion robots often also engage in more frequent interactions, suggesting the potential use of robot activity logs as objective indicators of human-AI attachment (Takada et al., 2023).

Further research is required to explore the generational differences and overall evolutionary trends in human-AI attachment. Generation Alpha, born in or after 2010, has grown up immersed in AI-driven technological environments and may exhibit higher levels of acceptance and attachment towards AI. Existing studies have discovered that children are more inclined to interact with robots and regard them as social partners (Burdett et al., 2022). However, comparative studies involving older adults and university students have produced contradictory results: older adults display a greater tendency to treat robots as social entities and report stronger attachment to virtual agents than college students do (Nomura and Sasa, 2009). Therefore, to control for confounding variables such as differences in cognitive abilities across age groups, future research should incorporate longitudinal designs to further investigate generational variations and society-wide shifts in human–AI attachment. Moreover, large-scale longitudinal studies would allow for the continuous tracking of societal levels of human-AI attachment, offering a more accurate assessment of the public’s acceptance of artificial intelligence and aiding in the identification of the current stage of AI technology development.

## 6 Conclusion

Human-AI Attachment (HAIA) can be conceptualized as a distinct subtype of parasocial attachment, characterized by a one-way, non-reciprocal emotional bond that uniquely incorporates direct interaction. This definition positions HAIA in a conceptual middle ground between traditional parasocial relationships, which lack interaction, and fully reciprocal interpersonal attachments.

HAIA begins with functional expectation, where positive anticipation of need fulfillment initiates engagement, often triggered by anthropomorphic design and shaped by pre-existing internal representations. This is followed by emotional evaluation, where the type of need fulfillment, the responsiveness of AI, and the individual’s ability to express needs generate positive affect. Ultimately, the establishment of representations occurs, where repeated experiences form stable internal working models of the AI and the self, developing HAIA styles that guide future interactions and create a feedback loop, continuously shaping the attachment bond.

## Data Availability

The original contributions presented in the study are included in the article/supplementary material, further inquiries can be directed to the corresponding author.
